# Model construction and validation of airflow velocity attenuation through pear tree canopies

**DOI:** 10.3389/fpls.2022.1026503

**Published:** 2022-11-08

**Authors:** Fubin Zhang, Hao Sun, Wei Qiu, Xiaolan Lv, Yunfu Chen, Guozhu Zhao

**Affiliations:** ^1^ College of Engineering/Key Laboratory of Intelligent Equipment for Agriculture of Jiangsu Province, Nanjing Agricultural University, Nanjing, China; ^2^ Institute of Agricultural Facilities and Equipment, Jiangsu Academy of Agricultural Sciences, Nanjing, China

**Keywords:** orchard, pear canopy, air-assisted spraying, airflow, resistance, modeling

## Abstract

To investigate the airflow velocity attenuation inside pear tree canopies and the factors that influence its effect on air-assisted spraying, the relationship between the resistance of the canopies to airflow and airflow velocity inside the canopies was determined. At the same time, the theoretical model of airflow velocity attenuation in the canopy was constructed, in which the velocity attenuation factor k and the incoming velocity were the model input values, and the airflow velocity in the canopy was the model output value. Then, experimental verification of the theoretical model was completed. The determination test of airflow velocity inside canopies with three leaf area densities revealed that the error range between the established theoretical model and the experimental airflow velocity inside the pear tree canopy was 0.11–1.25 m/s, and the mean size of the model accuracy was 83.4% under various working conditions. The results revealed that the region from a depth of 0 m to 0.3 m inside the canopy was the rapid attenuation area of the airflow and that from 0.3 m to 0.9 m was the low attenuation area. Furthermore, they revealed that high-speed airflow could strongly disturb the outer branches and leaves, greatly changing the windward area of the canopy blades and thus affecting the accuracy of the model. By introducing a dynamic parameter of the canopy leaf windward area for model correction, the R^2^ of the model was above 0.9. Finally, validation of the model was performed in an air-assisted spraying operation in an orchard. This study can provide a theoretical basis for the regulation of airflow parameters of air-assisted spraying of pear trees.

## 1 Introduction

As the third largest cultivated fruit tree in China, the pear tree can generate large economic benefits for China every year; however, it is prone to pests and diseases during the growth process. As they prevent the yield loss caused by pests and diseases, pesticides are an indispensable preventive measure in the process of pear tree cultivation (Pan et al., 2019; Li et al., 2022). However, frequent chemical application will cause problems such as pesticide residues and serious pollution.

Air-assisted spraying technology delivers atomized liquid to a canopy with the help of high-speed airflow, which helps to achieve a more homogeneous distribution and significantly improves the deposition of droplets and is, therefore, one of the most important technical measures for increasing the efficiency and reducing the application of pesticides in orchards (Planas et al., 2002; Panneton et al., 2005). Presently, researchers have conducted several studies on air-assisted application technology, mostly focusing on changing the type of fan or related parameters to investigate the movement of airflow in the air (Mion et al., 2011; Chao et al., 2019; Qiu et al., 2020). However, the presence of canopy branches and leaves in fruit trees inevitably makes airflow movement in the canopy different from that in the air. Therefore, researchers pay more and more attention to the study of canopy airflow attenuation. Some scholars have established artificial canopy (Musiu et al., 2019) and computational fluid dynamics (CFD) porous-medium canopy models (Endalew et al., 2008; Salcedo et al., 2017; Hong et al., 2018) to explore the relationship between canopy airflow attenuation and canopy density. Although the above method can reflect the energy change of the airflow passing though the canopy to a certain extent, and has a certain enlightening effect on the study of airflow attenuation in the fruit trees canopy, the actual fruit-tree canopy structure is relatively complex, and there are certain limitations in establishing artificial canopy and CFD models.

The above research shows that scholars have transitioned from focusing on the characteristics of sprayer to the stage of mechanical integration focusing on the characteristics of the canopy, and have paid attention to the influence of the canopy on airflow attenuation. However, there are still some limitations in the study of airflow attenuation in the canopy, mainly because the energy changes of airflow through the canopy are complex, and the characteristics of the canopy will significantly affect the airflow resistance and attenuation in the canopy (Walklate, 1992). Currently, Fruit trees still face “excessive deposition of droplets on the outside of the canopy and insufficient deposition of droplets in the inner chamber and the back of the leaves, the pest-prone areas” (Gil Sierra et al., 2006; Javier Garcia-Ramos et al., 2012; Dekeyser et al., 2014). Therefore, it is important to consider the airflow through a canopy and clarify its attenuation pattern to further improve the application effect and reduce the amount of liquid spray.

For that reason, this study took the crown pear as the research object and constructed a relational quantitative model for the attenuation of intra-canopy airflow velocity and based on the results of previous research on intra-canopy airflow velocity; then, a three-dimensional air-assisted resistance experimental platform was built to measure the relevant parameters using the pear tree canopy as the experimental sample. Furthermore, the experimental values of intra-canopy airflow velocity were obtained and compared with the theoretical values to verify the accuracy of the model. The model errors were then analysed and corrected for accuracy. Finally, the applicability of the model in air-assisted spraying operation was verified in a real orchard. This study aims to clarify the attenuation pattern of airflow velocity inside a canopy and its influencing factors in the air-assisted spraying operation and provide a new basis for the machinery and parameter setting of air-assisted application in orchards.

## 2 Materials and methods

### 2.1 Airflow velocity attenuation model

#### 2.1.1 Theoretical model

The air flow is gradually weakened by the obstructive effect of branches and leaves as it flows inside the canopy. According to Кайгородoв (Fu, 1963),


(1) $$\begin{array}{c} dv=-kvdy. \end{array}$$

The variation in Eq. (1) is integrated in  *v*:v_*_→*v*(*y*);   *y*:0→*y* :


(2) $$\int_{v_{*}}^{v\left(y\right)}\frac{1}{v}dv=\int_{0}^{y}-kdy.$$

Thus,


(3) $$\begin{array}{c} v\left(y\right)=v_{*}e^{-ky}, \end{array}$$

*v*(*y*): the velocity of the airflow at different depths inside the canopy [m/s];*v_∗_*: the velocity of the airflow when it reaches the surface of the canopy, i.e., the incoming velocity [m/s];*k*: the velocity attenuation factor; *y*: the depth from the canopy surface [m].

#### 2.1.2 Determination of the velocity attenuation factor *k*

Among them, the velocity attenuation factor *k* is influenced by the canopy structure and other factors. Thus, this study analyzed the causes of airflow attenuation in the canopy, clarified the factors that lead to the change in *k* values, constructed a correlation model between the *k* values and canopy structure parameters, and finally obtained a theoretical model of airflow attenuation in the canopy that can guide pesticide application.

The fundamental reason for the attenuation of airflow velocity inside the canopy is that when airflow passes inside the canopy, the airflow collides with the branches and leaves of the canopy and is obstructed by the branches and leaves, i.e., the drag effect, which generates the attenuation of airflow velocity inside the canopy. Thus, to obtain the relationship between the velocity attenuation factor and the canopy structure, it is necessary to investigate the resistance of the canopy to the airflow so that *k* values can be analyzed by substitution to clarify the variation pattern of the velocity attenuation factor and the mechanism of action leading to the velocity change.

According to the Burr effort equation, when an impermeable object is placed at a flow field with hydrostatic pressure of *P*_0_, air density of *ρ*, and flow velocity of *v*, and when the flow velocity at the leeward edge of the object drops to 0 and the static pressure becomes *P*, there is a dynamic pressure of


(4) $$\begin{array}{c} P-P_{0}=\frac{1}{2}\rho v^{2}. \end{array}$$

The resistance of the canopy to the airflow is calculated by the air resistance equation based on the relationship between the dynamic pressure and drag:


(5) $$\begin{array}{c} F=\frac{1}{2}C_{d}\rho v^{2}S_{0}. \end{array}$$

F: the canopy resistance to airflow [N]; *C_d_*: the air resistance coefficient; *ρ*: the air density [kg/m^3^]; *v*: the airflow velocity [m/s]; *S*_0_: the windward area [m^2^].

At any point inside the canopy (*x, y, z*) where the velocity of the airflow is *v*(*x, y, z*), take a volume element *dV* and let the windward area per unit volume of the canopy be *T*, it is a plane perpendicular to the airflow, so the air resistance coefficient *C_d_* is 1.0 (Wu and Qian, 2017). Then, the windward area of the leaves in the volume *dV* is *TdV*. Therefore, the resistance to the airflow in the volume *d*V is


(6) $$\begin{array}{c} dF=\frac{1}{2}\rho {\left[v\left(x,y,z\right)\right]}^{2}TdV, \end{array}$$

(7) $$\begin{array}{c} dV=dxdydz, \end{array}$$

*T*: the windward area per unit volume of the canopy [m^2^]; *v(x, y, z)*: the velocity at any point within the canopy [m/s]; *V*: the volume of the canopy [m^3^].

For this experiment, the experimental range is in the circular air-assisted area when the air blown from the fan reaches the canopy surface. The radius of the air-assisted area is *R*, and the canopy thickness is *D*. Therefore, the area size is


$$x:-R\to R;\;y:0\to D;\;z:-\sqrt{R^{2}-x^{2}}\to \sqrt{R^{2}-x^{2}}.$$

The resistance to airflow created by the canopy in the air-assisted area is


(8) $$F=\int_{-R}^{R}\int_{0}^{D}\int_{-\sqrt{R^{2}-x^{2}}}^{\sqrt{R^{2}-x^{2}}}\frac{1}{2}\rho {\left[v\left(x,y,z\right)\right]}^{2}TdV.$$

In the above equation, as the airflow is generated by a fan, the airflow velocity in the direction perpendicular to the horizontal depth is small; therefore, only the airflow velocity along the y direction is retained:


(9) $$\begin{array}{c} v\left(x,y,z\right)=v\left(y\right), \end{array}$$

And


(10) $$\begin{array}{c} F=\frac{\pi R^{2}T\rho {\text{v*}}^{2}}{4k}\left(1-e^{-2kD}\right). \end{array}$$

The above equation constructs an expression for the airflow resistance when the airflow passes through the canopy of pear trees. The windward area per unit volume of the canopy in the equation cannot be obtained directly and needs to be further transformed.

**Figure 1:**
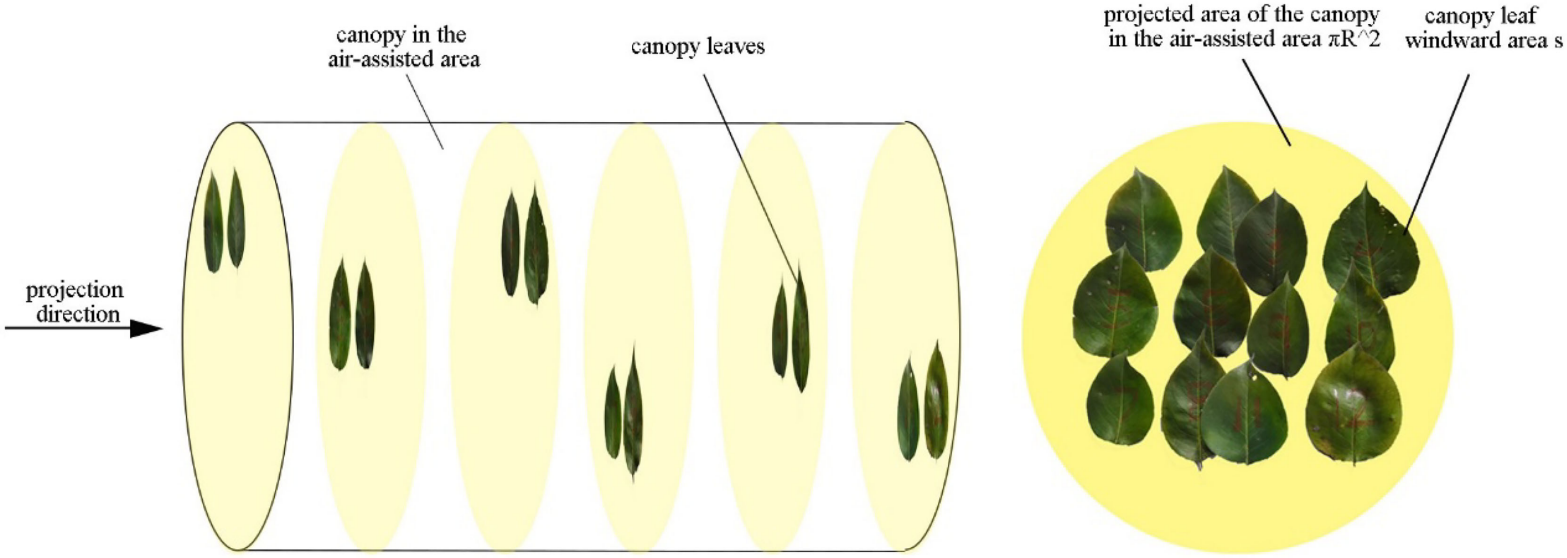
Schematic of the canopy projection.

In this study, image processing (**Figure 1**) was used to obtain the ratio of the canopy leaf windward area to its projected area in the air-assisted area. Then, the windward area per unit volume of the canopy was obtained by Eq. (11).


(11) $$\begin{array}{c} T=\frac{s}{\pi R^{2}D^{\prime }}, \end{array}$$

*πR* ^2^ : the projected area of the canopy in the air-assisted area [m^2^];  *D* ^′^ : the thickness of a section of canopy branches and leaves [m], *s*: the windward area of the canopy leaves [m^2^].

Substituting the windward area per unit volume of the canopy, i.e., Eq. (13) into Eq. (12), we obtain


(12) $$\begin{array}{c} F=\frac{s\rho {\text{v*}}^{2}}{4kD^{\prime }}\left(1-e^{-2kD}\right). \end{array}$$

The parameters such as flow resistance and windward area of the leaves in the above equation can be determined experimentally; therefore, the velocity attenuation factor *k* can be determined from Eq. (12), and the theoretical model of airflow velocity attenuation inside the canopy can be obtained after substituting Eq. (12) into Eq. (3).

### 2.2 Measurement of airflow velocity attenuation model parameters

When measuring the canopy aerodynamic resistance and other related parameters, it is difficult and time-consuming to use the whole pear tree for experimental measurement. In order to ensure the accuracy of the test results, pear tree branches and leaves with different leaf area densities were used for the test. For minimizing the influence of external airflow on the test results, the test was carried out in the laboratory without wind. In September 2021, fresh samples of pear tree branches and leaves were collected from the family farm of Sisi Yu in Pukou District, Nanjing, China for the canopy simulation test in a laboratory environment. Seven-year-old Crown pear trees with an average tree height of 2.1 m, average canopy width of 1.2 m, and average stem height of 0.7 m were tested. The leaf area density of the pear canopy in the orchard ranged from 3.94 –5.72 m^2^/m^3^, the leaves of the main branches were kept in their natural form, and the time between sampling and conducting the experiment was controlled to be within 6 hours.

#### 2.2.1 Test platform

**Figure 2:**
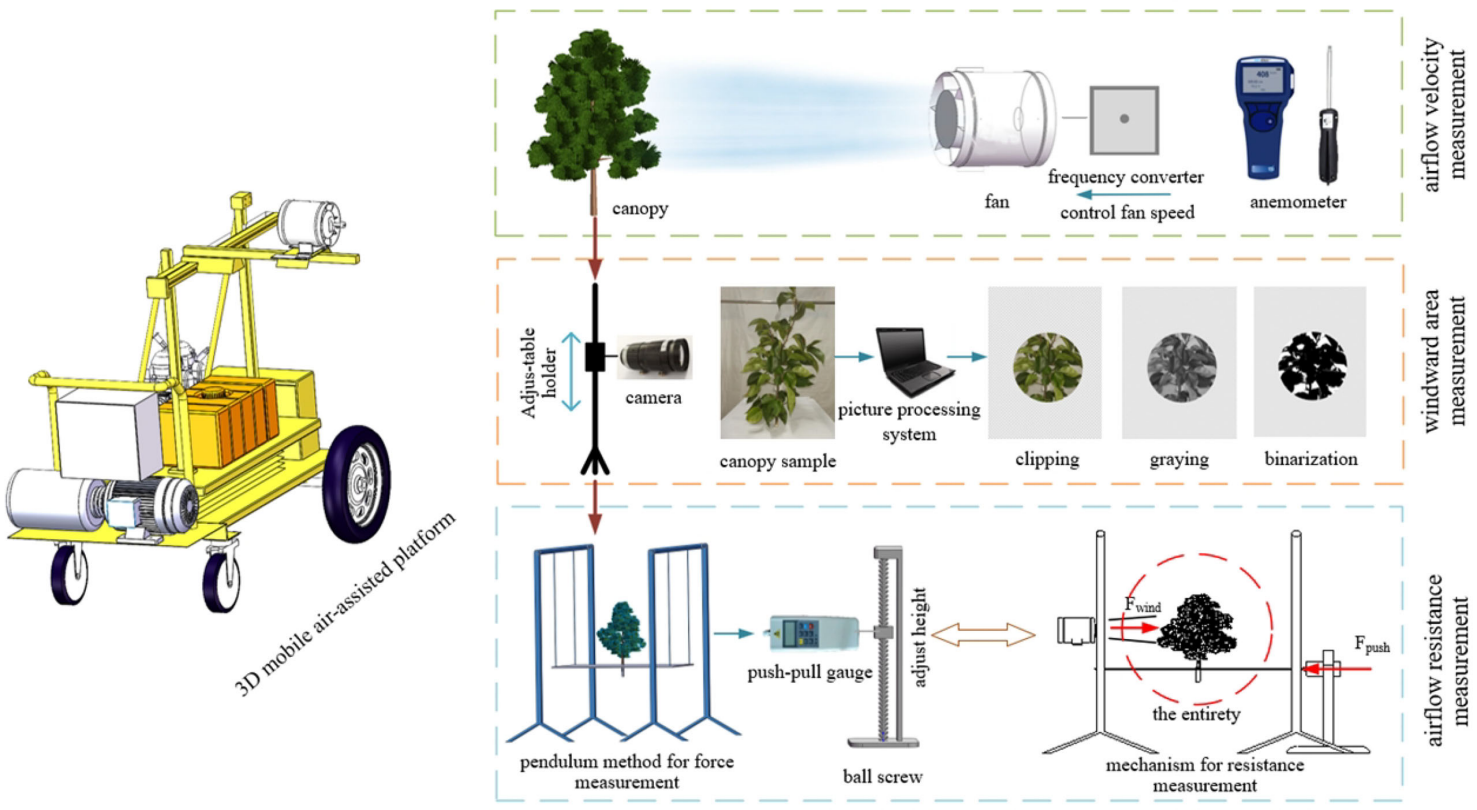
Experimental system.

The experimental system consisted of two main components: the air-assisted platform and the measurement system (**Figure 2**). The air-assisted platform consisted of a variable frequency mixed-flow duct fan (diameter: 260 mm; air flow: 1080 m^3^ h^-1^; rated speed: 3200 r min^-1^), mobile working platform (at the fixed walking speed of 1 m/s), and fan position adjustment system (0–1 m range in the walking direction; 0–1 m range in the air delivery direction; 0–1.2 m range in the vertical direction). The fan speed was controlled by a frequency converter. The measurement system consisted of an TSI 9565 anemometer(TSI Inc., Minnesota, USA, measurement error of ±  0.025 m/s), USB HD camera s(shooting speed: 110 fps); data storage device, portable computer and push-pull gauge (ELECALL, China, Zhejiang, the maximum load value was 10 N, the load division was 0.001 N, and the indication error was ± 0.5%).

#### 2.2.2 Measurement of airflow velocity, leaf windward area and airflow resistance in canopy

**Figure 3:**
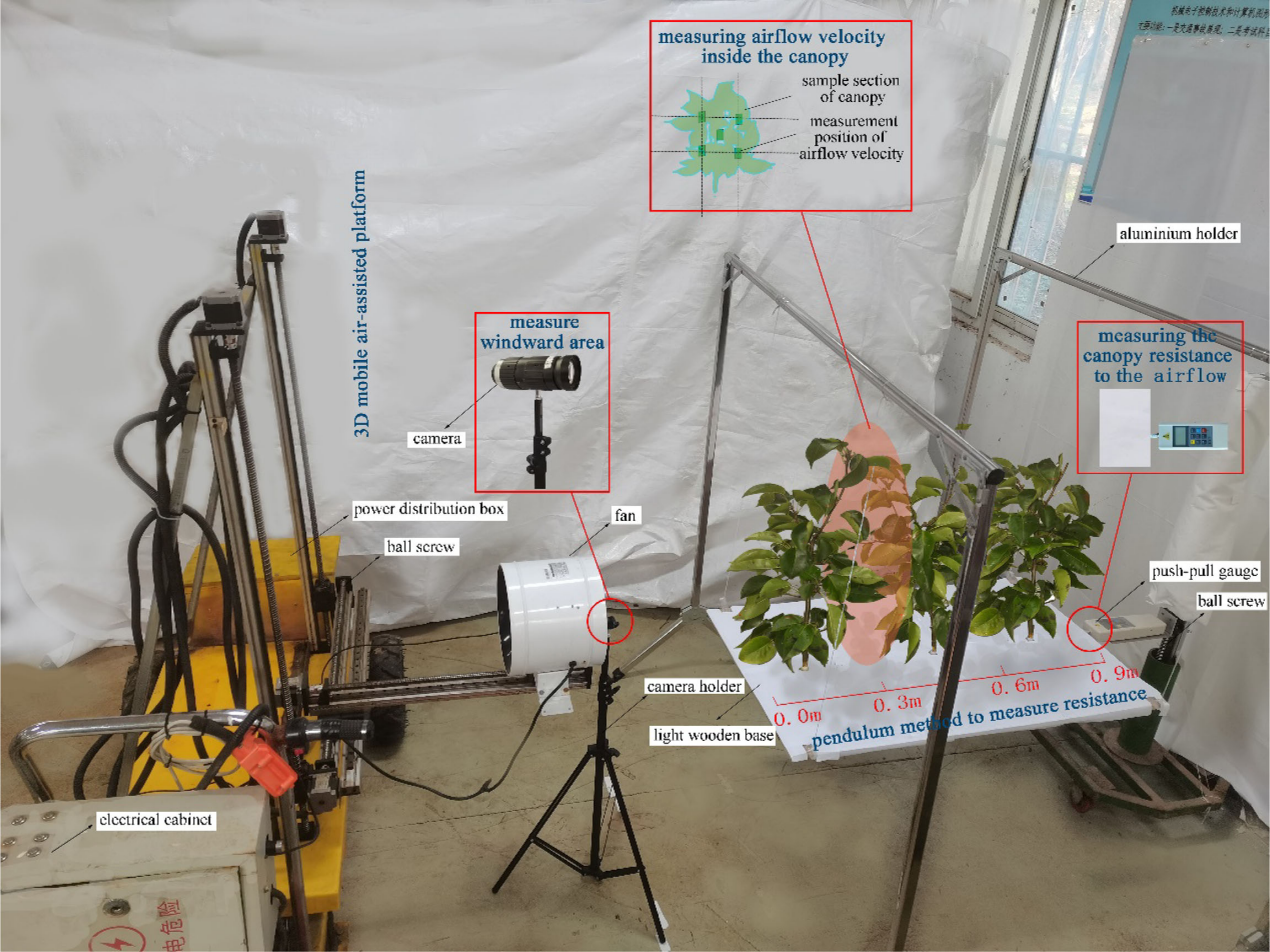
Experiment set-up.

The test is shown in **Figure 3**. For the measurement of airflow velocity, the airflow velocity reaching the canopy surface and the airflow velocity at different depths in the canopy were measured by the TSI 9565 anemometer.

For the canopy leaf windward area: a high-speed camera was used to take pictures of the canopy in the direction of the airflow, with a white cloth as the background to avoid the influence of other debris. In the windless condition, the size of the air-assisted area on the canopy was marked with a marker as a selection range for the image processing later. Canopy photos of each leaf area density were taken to calculate the windward area of the canopy leaves in the air-assisted area. Combined with the volume of the canopy in the air-assisted area, the windward area per unit volume of the canopy was obtained for the canopies of different leaf area density.

For the canopy resistance to the airflow: the 3D mobile air-assisted platform was adjusted by turning the ball screw to move the fan to a position at the height of the middle of the canopy, with the air outlet position being 0.5 m from the canopy surface. We found that the airflow blown by the duct fan made a circle of radius 0.16 m after reaching the air-assisted area of the canopy surface. The height of the force measurement platform was adjusted to make the force gauge level with the base plate, bringing it against the base plate and setting the pressure to zero before the experiment started. The fan was started, and the fan wind speed knob was adjusted to control the airflow velocity of reaching the canopy surface. The push-pull gauge was then used to measure the canopy resistance to the airflow.

### 2.3 Verification of airflow velocity attenuation model

According to previous studies (Guan, 1998), the windward area per unit volume of the canopy is proportional to the leaf area density; therefore, this study set up three different leaf area densities of pear canopies to carry out the experiments: a sparse canopy with a leaf area density of 4.15 m^2^/m^3^, medium canopy with a leaf area density of 4.79 m^2^/m^3^, and compact canopy with a leaf area density of 5.65 m^2^/m^3^.

The canopy of 0.9 m-depth was selected for verification test., The experimental values of the airflow velocity within the canopy at heights of 0.15 m, 0.30 m, 0.45 m, 0.60 m, 0.75 m, and 0.90 m for incoming flow velocities of 4 m/s, 8 m/s, and 12 m/s were detected. At the same time, the leaf windward area of pear canopy and the canopy resistance under different airflow velocity were measured, and the theoretical value of canopy airflow velocity was calculated by formulas (12) and (3).

The theoretical values of the airflow velocity in the canopy were compared with the experimental values for analysis to verify the accuracy of the model and the correction model. The errors and model accuracy were calculated as follows.


(13) $$\begin{array}{c} v_{w}=|v_{2}-v_{1}| \end{array}$$

(14) $$\begin{array}{c} I=\left(1-\frac{|v_{2}-v_{1}|}{v_{1}}\right)\times 100\% \end{array}$$

*v_w_*: the error between the experimental and theoretical values of airflow velocity [m/s]; *v*_2_: the experimental value of airflow velocity [m/s]; *v*_1_: the theoretical value of airflow velocity [m/s]; *I*: the model accuracy.

### 2.4 Field test

**Figure 4:**
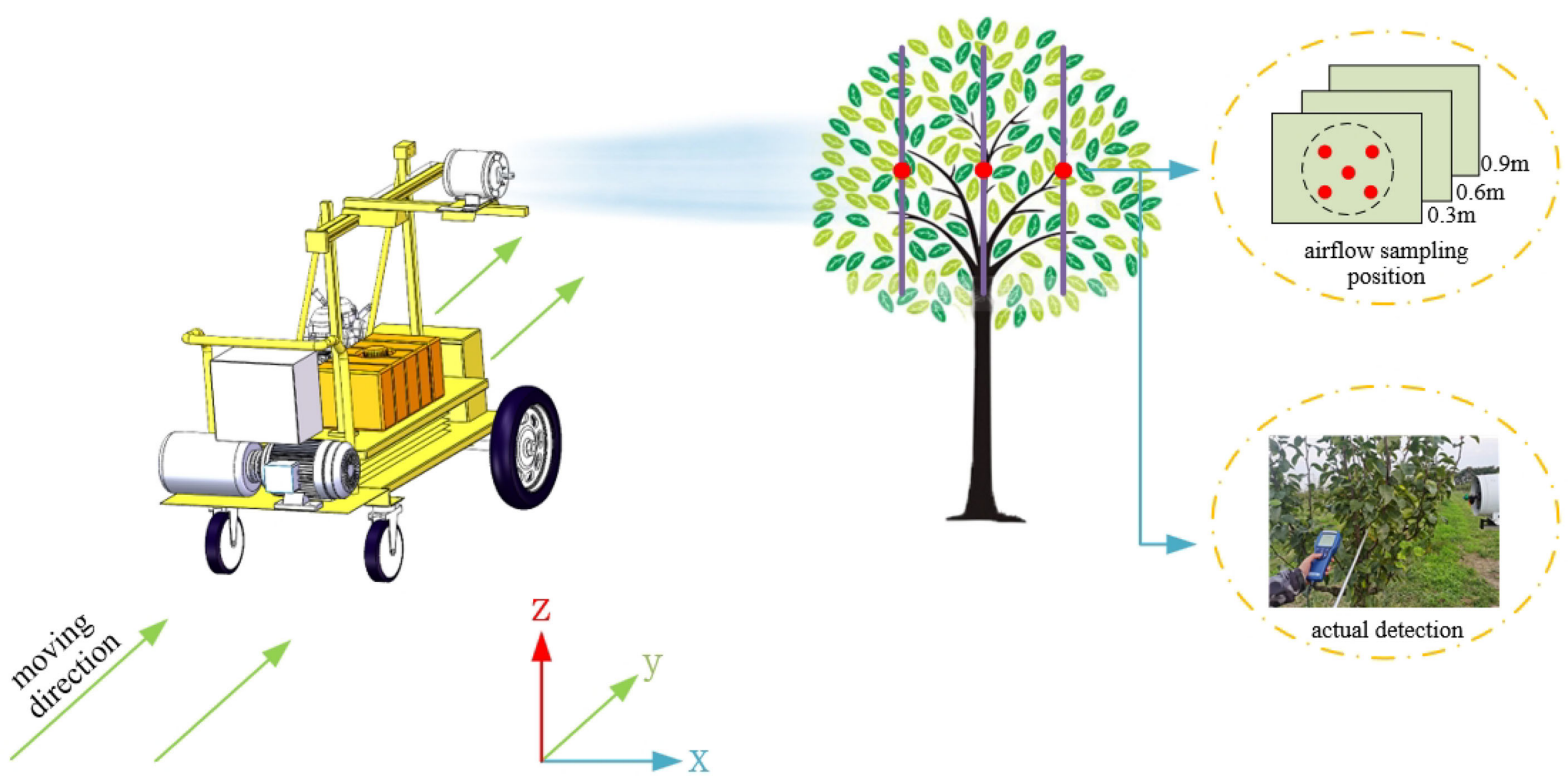
Airflow velocity measurements inside the canopy of pear trees in an orchard.

To further verify the applicability of the intra-canopy airflow velocity attenuation model in the field environment, A field test was conducted in the crown pear orchard in the Yu Sisi Family Farm (118.42°E, 32.98°N) in Nanjing in June 2022(average external temperature: 28°; average airflow velocity: 0.3 m/s). Three pear trees with leaf area densities similar to those used in the laboratory test were selected: pear tree A with a leaf area density of 4.08 m^2^/m^3^; pear tree B with a leaf area density of 4.72 m^2^/m^3^; and pear tree C with leaf area density of 5.58 m^2^/m^3^. Two sets of intra-canopy airflow velocity measurements were conducted in this orchard experiment, and the data acquisition method is shown in **Figure 4**: (1) the fan speed knob was turned to ensure the inflow velocity was 8 m/s and 12 m/s when the inflow reached the surface of the pear tree canopy [the optimum airflow parameter in the field is 9-11 m/s (Dai, 2008)], and the moving speed of the experimental platform was set to 0 m/s to measure the airflow velocity at the positions of 0.30 m, 0.60 m and 0.90 m inside the pear tree canopy with three different leaf area densities; (2) the fan speed knob was turned to ensure the inflow velocity was 8 m/s and 12 m/s when the inflow reached the surface of the pear tree canopy, and the moving speed of the experimental platform was set to 1 m/s to measure the airflow velocity at the positions of 0.30 m, 0.60 m and 0.90 m inside the pear tree canopy for three different leaf area densities.

## 3 Results

### 3.1 Calculation of the velocity attenuation factor k

In this study, the canopy airflow resistance and canopy leaf windward area were measured in a laboratory setting for three different leaf area densities at incoming velocities of 4 m/s, 8 m/s and 12 m/s, as shown in **Table 1**.

**Table 1:** Canopy leaf windward area values and canopy resistance to airflow values.

Leaf area density (m^2^ /m^3^)	Windward area of canopy Leaves in air-assisted area *s* (m^2^)	Windward area per unit Volume of the canopy *T* (m^2^ /m^3^)	Resistance F (N)		
			Incoming Flow speed v_*_ (4 m/s)	Incoming Flow speed v_*_ (8 m/s)	Incoming Flow speed v_*_ (12 m/s)
4.15	0.057	2.353	0.97	2.96	4.98
4.79	0.060	2.480	0.94	2.63	4.49
5.65	0.065	2.673	0.88	2.35	4.11

v_*_ is the velocity of the airflow when it reaches the surface of the canopy.

The data in **Table 1** can be collated and calculated by Eq. (14) to obtain the velocity attenuation factor *k* for canopies of different leaf area densities at different incoming flow velocities, as shown in **Table 2**.

**Table 2:** Velocity attenuation factor at different operating conditions.

Incoming flow velocity *v*_∗_ (m/s)	Speed attenuation factor *k*		
	Sparse canopy(Lr = 4.15 m^2^/m^3^)	Medium canopy(Lr = 4.79 m^2^/m^3^)	Compact canopy(Lr = 5.65 m^2^/m^3^)
4	0.746	0.874	1.087
8	1.159	1.457	1.824
12	1.682	2.014	2.401

*v*_∗_ is the velocity of the airflow when it reaches the surface of the canopy.

### 3.2 Validation of the airflow velocity attenuation model

**Figure 5:**
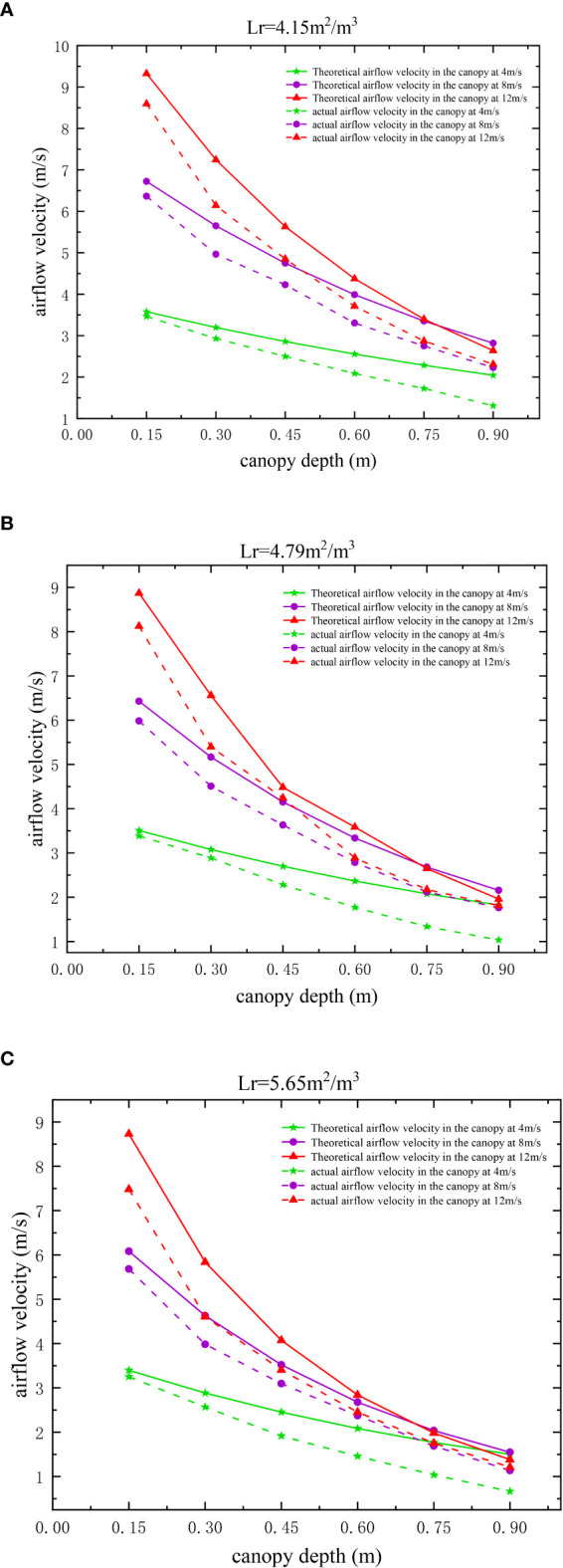
Variation in the intra-canopy airflow velocity with canopy depth for different leaf area densities. **(A–C)**.

The experimental and theoretical airflow velocities at positions 0.15 m, 0.30 m, 0.45 m, 0.60 m, 0.75 m, and 0.90 m in the canopy for three different leaf area densities are given in **Figure 5**. The processed data reveal that the error values of the theoretical and experimental airflow velocities at different positions in the canopy for the three different leaf area densities at each incoming velocity range from 0.11 to 1.25 m/s, while the mean theoretical model accuracy is 83.4% for all the tested operating conditions. Among them, at an incoming flow velocity of 4 m/s, for the pear canopy with leaf area densities of 4.15 m^2^/m^3^, 4.79 m^2^/m^3^ and 5.65 m^2^/m ^3^, the mean model accuracies of the attenuation of airflow velocity in the canopy are 82.8%, 78.5%, 72.7%, respectively; at the incoming flow velocity of 12 m/s, for leaf area densities of 4.15 m^2^/m^3^, 4.79 m^2^/m^3^ and 5.65 m^2^/m^3^, the mean accuracies of the intra-canopy airflow velocity attenuation model are 86.7%, 87.3%, and 85.3%,respectively, for the pear canopy. The model accuracies lie in between the values above at the incoming velocity of 8 m/s. In summary, with an increase in airflow velocity, the mean value of model accuracy increases. At smaller incoming velocities, although the accuracy (relative difference) is lower, the absolute difference is in the range of 0.11–0.84 m/s, which meets the requirements of model accuracy.

### 3.3 Error analysis and correction of the airflow velocity attenuation model

**Figure 6:**
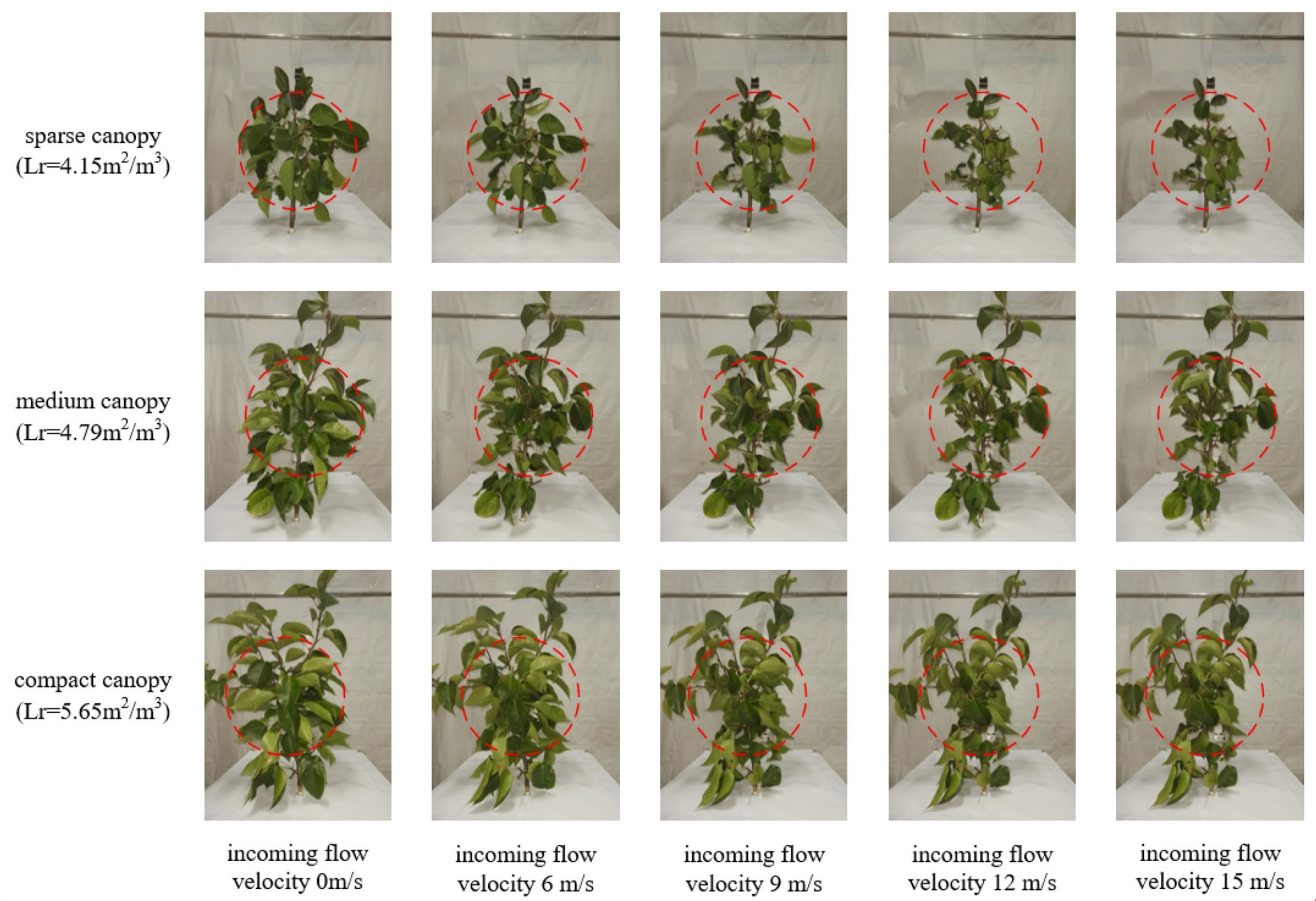
Variation in the windward area of the canopy leaves for different incoming flow velocities.

An analysis of the results in **Figure 5** reveals that the theoretical values of the airflow velocity in the canopy obtained using the model in this paper are larger than the experimental values, and the error range is between 0.11–1.25 m/s. This is because the model calculates the theoretical airflow velocity using the windward area of the canopy leaves measured in the windless state, yet the leaf windward area of 0–0.3 m in the canopy in the experiment greatly varies with an increase in the incoming flow velocity, resulting in large errors between the theoretical and experimental airflow velocities, as shown in **Figure 6**

**Figure 7:**
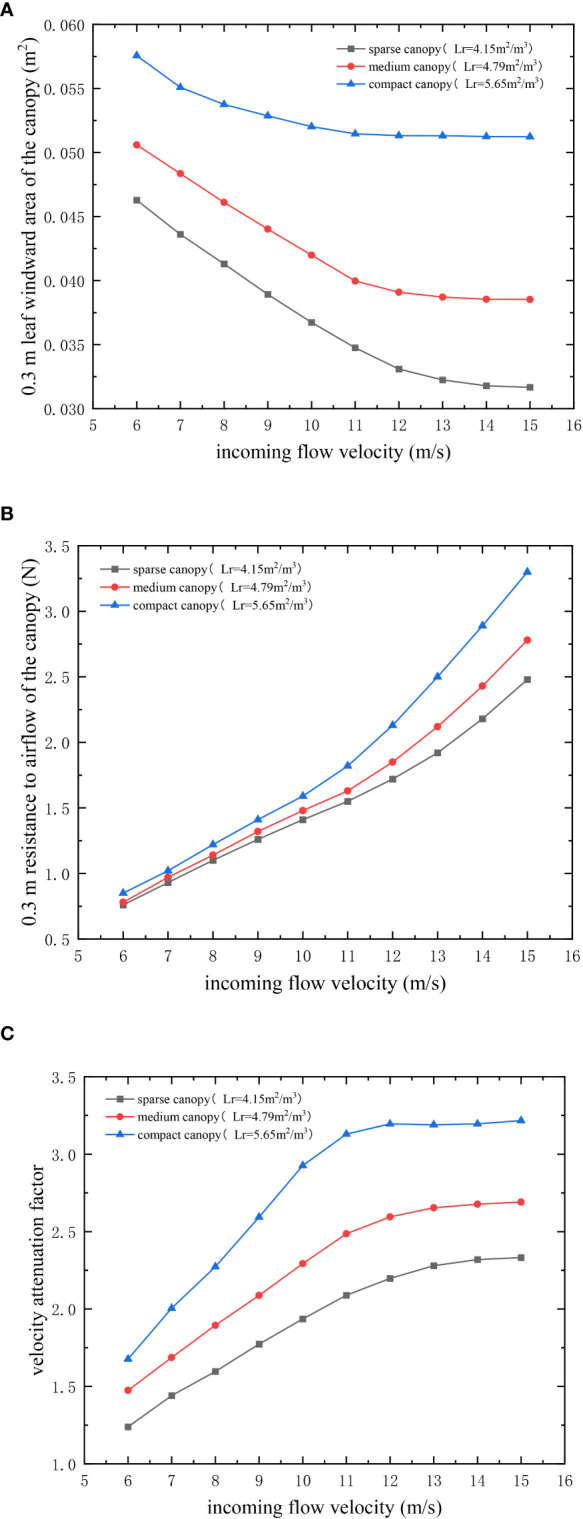
Leaf windward area of the canopies and velocity attenuation factor versus the incoming flow velocity. **(A–C)**.

To investigate the specific causes of such errors and the variation law, the dynamic leaf windward area and canopy resistance to airflow for a 0.3 m-thick canopy at an incoming velocity of 6–15 m/s were measured by the method of determining the canopy aerodynamic parameters in Section 2.2. The variation in the leaf windward area, canopy resistance to airflow, and velocity attenuation factor for a 0.3 m-thick canopy at different incoming velocities were clarified. The relationship is shown in **Figure 7**. It can be seen that the incoming airflow velocity is negatively correlated with the canopy leaf windward area (**Figure 7A**), positively correlated with the resistance (**Figure 7B**) and positively correlated with the velocity attenuation factor (**Figure 7C**). When the incoming velocity increases, the leaves at 0–0.3 m in the canopy are converged by the airflow, the windward area of the canopy leaves reduces, and the velocity attenuation increases. The experimental airflow velocity is smaller than the theoretical airflow velocity calculated using the static canopy leaf windward area, and with an increase in the incoming flow velocity in a certain range, the error values between the two expand. When the incoming velocity increases to a certain degree (Lr = 4.15 m^2^/m^3^ corresponds to 14 m/s; Lr = 4.79 m^2^/m^3^ corresponds to 12 m/s; and Lr = 5.65 m^2^/m^3^ corresponds to 11 m/s), the elastic deformation of the canopy leaves reaches its maximum value, and the canopy leaf windward area and velocity attenuation factor stabilize (Molina-Aiz et al., 2006). The increase in the error between the experimental and theoretical values of the airflow velocity also stabilizes.

In summary, in order to improve the model accuracy, the dynamic changes in the canopy leaf windward area based on different incoming flow velocities were used to modify the 0–0.3 m canopy airflow resistance model. The modified model is shown in Eq. (15). The corresponding canopy leaf windward area and canopy resistance to airflow at different incoming flow velocities in **Figure 7** were substituted into the model, and the theoretical values of airflow velocity in the 0.3 m canopy were calculated and compared with the experimental values of airflow velocity to verify the accuracy of the revised model.


(15) $$\begin{array}{c} \{\begin{array}{c} F=\frac{s^{0}\rho {\text{v*}}^{2}}{4kD^{\prime }}\left(1-e^{-2kD}\right)\;\;\;\;\;\;\;\;\;\;\;\;\;\;\;\;\left(D>0.3m\right)\\ F=\frac{s^{*}\rho {\text{v*}}^{2}}{4kD^{\prime }}\left(1-e^{-2kD}\right)\;\;\left(0m\leq D\leq 0.3m\right) \end{array} \end{array}$$

*s* ^0^: the windward area of the canopy leaves in the windless condition [m^2^]; *s* ^*^: the dynamic canopy leaf windward area at different incoming flow velocities [m^2^].

**Figure 8:**
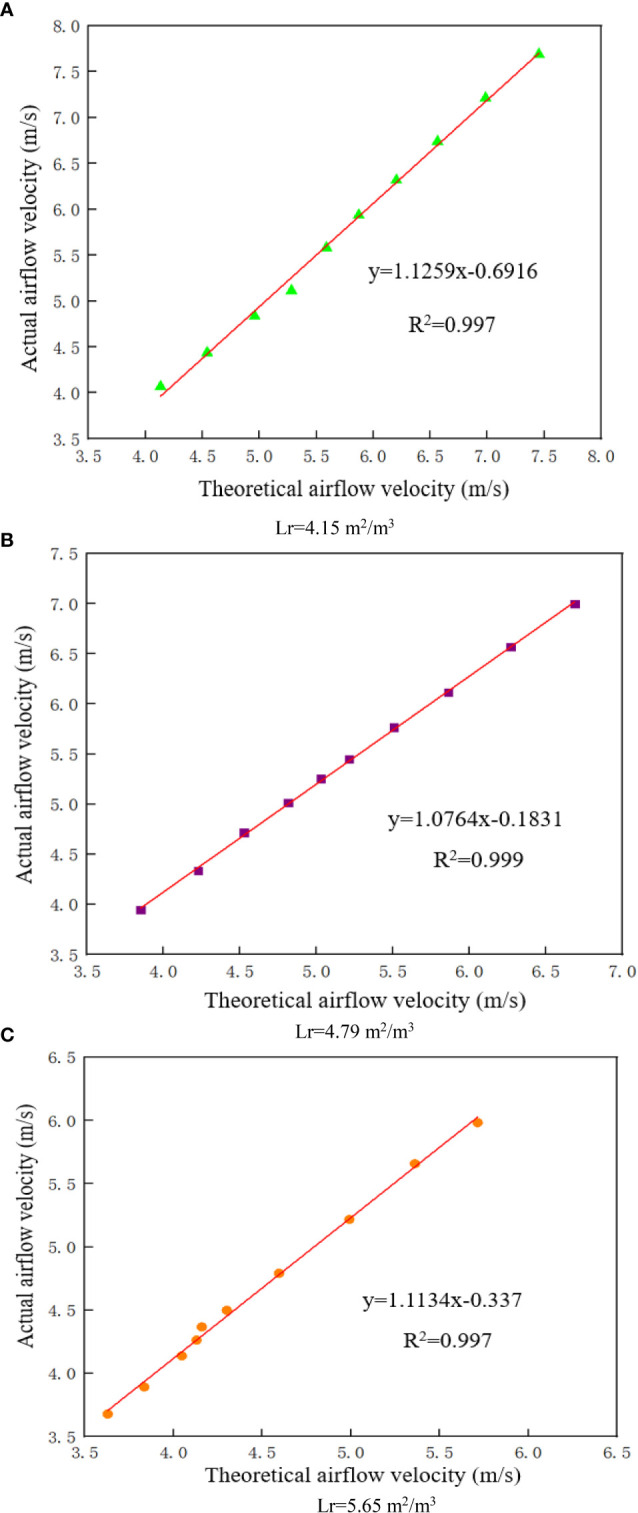
Airflow velocity at a depth of 0.3 m in the canopy **(A)** Lr=4.15 m2/m3 **(B)** Lr=4.79 m2/m3 **(C)** Lr=5.65 m2/m3.

The experimental and theoretical airflow velocities at the 0.3 m canopy at an incoming flow velocity of 6–15 m/s were calculated and perform a linear regression analysis, as shown in **Figure 8**. The processed data in the figure reveal that, for the three different leaf area densities in this study, the R^2^ of the theoretical and experimental airflow velocity at the depth of 0.3 m in the canopy is above 0.9 even when the incoming flow velocity is large, as shown in (**Figure 8A–C**). This indicates that the theoretical airflow velocity is in good agreement with the actual airflow velocity. It can be seen that the modified model, which considers the coupling effect between the airflow and canopy and calculates the dynamic values of canopy leaf windward area has largely solved the problem of large errors between theoretical and experimental values of airflow velocity in the outer layer of the canopy when the incoming flow velocity is large.

### 3.4 Analysis of field test results

**Figure 9:**
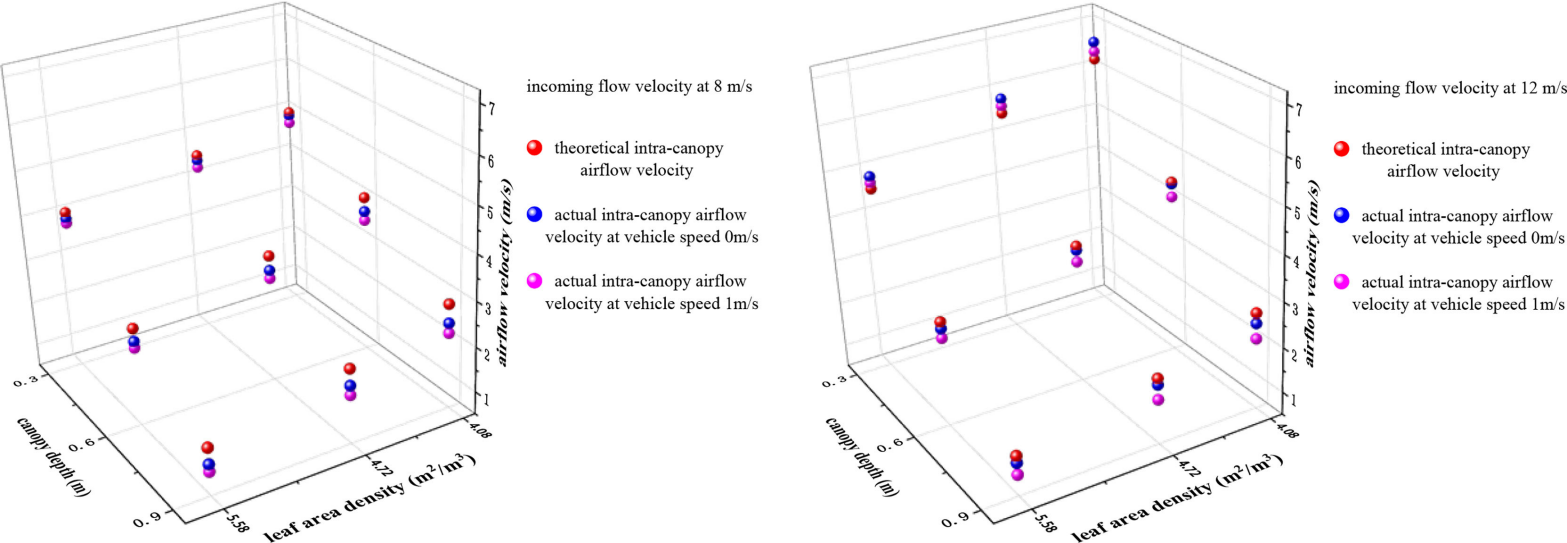
Airflow velocities in the pear canopy at different working conditions.

In this study, two sets of experiments were designed to determine the airflow velocity inside the pear canopy in an orchard environment, where the incoming flow velocities are 8 m/s and 12 m/s, respectively, and the vehicle speeds are 0 m/s and 1 m/s. The results were compared and analyzed with the modified theoretical airflow velocities inside the canopy, as shown in **Figure 9**.

(1) The effect of the vehicle speed on the magnitude of intra-canopy airflow velocity was investigated by setting the vehicle speeds to 0 m/s and 1 m/s. When the incoming flow velocity was 8 m/s, the mean relative errors at the positions of 0.3 m, 0.6 m and 0.9 m in the canopy were 3.2%, 5.8% and 11.4%, respectively; when the incoming flow velocity was 12 m/s, the mean relative errors at the positions of 0.3 m, 0.6 m and 0.9 m were 2.9%, 7.3%, and 17.9%, respectively. For the three different leaf area densities, the errors of the experimental values of the airflow velocity at any position in the canopy were within 0.4 m/s at incoming flow velocities of 8 m/s and 12 m/s.

Affected by the vehicle speed, the central region of the airflow is slightly off the measurement point of the canopy when the machine reaches the measurement location, resulting in a mildly smaller experimental airflow velocity as the vehicle moves. The difference increases slightly with the depth of the canopy, but remains small in general.

(2) Regarding the applicability of intra-canopy airflow velocity attenuation model in an orchard environment, a comparison between the theoretical and experimental values of the intra-canopy airflow velocities of the pear trees at a vehicle speed of 1 m/s was conducted. It was found that the average model accuracies at the positions of 0.3 m, 0.6 m and 0.9 m were 94.4%, 85.4% and 72.3%, respectively, when the incoming flow velocity was 8 m/s. When the incoming flow velocity was 12 m/s, the average accuracy at the position of 0.3 m, 0.6 m and 0.9 m were 97.2%, 89.3% and 75.1%, respectively. The errors between the theoretical and experimental values of the airflow velocity at any position within the canopies of the three different leaf area densities at the incoming flow velocities of 8 m/s and 12 m/s were within the range of 0.7 m/s.

Only the attenuation effect of the canopy on the airflow was considered when using the model for the theoretical airflow velocity solution; however, the experimental results revealed that the airflow was also obstructed by the air (The deeper the canopy, the stronger the cumulative influence of the air). Therefore, the accuracy of the model was between 70–80% at the canopy position of 0.9 m, but the absolute difference was only between 0.4 and 0.7 m/s. In summary, the attenuation model of intra-canopy airflow velocity constructed in this study is equally applicable in air-assisted spraying in orchards.

## 4 Discussion

The superiority of air sprayers in orchard plant protection operations and their control effects have been discussed for decades (Delele et al., 2007). Airflow is one of the most essential parameters of air-spray technology. Therefore, clarifying the attenuation of airflow in a canopy is key to achieving the regulation of airflow parameters of wind-delivered spraying in orchards, and promoting the effect of pest control.

Previous studies on airflow attenuation have reported that the canopy of fruit trees exerts resistance to the flow of air through the passage and captures kinetic energy to reduce the flow rate (Endalew et al., 2008; Endalew et al., 2009). Hence, CFD simulation models were constructed based on resistance coefficients, leaf area density, and other parameters to analyse canopy airflow (Yue et al., 2007; Zhan et al., 2017). However, it is difficult to theoretically reveal the airflow attenuation in the canopy and its influencing factors this way. Based on previous research, the paper constructs a airflow velocity attenuation model in the canopy. The experimental results in this study can reveal the effects of the three variables of leaf area density, canopy depth and incoming velocity on the attenuation of airflow velocity in the canopy respectively: (1) For the canopy with a large leaf area density, the attenuation effect of the canopy on airflow velocity is stronger owing to the denser branches and leaves, and stronger obstruction effect in the canopy; (2) with an increase in the canopy depth, the attenuation degree of airflow velocity gradually decreases, with a rapid attenuation zone at a height of 0–0.3 m and a slow attenuation zone at a height of 0.3–0.9 m. It can be found that the attenuation of airflow in the canopy mainly occurs in the outermost layer of the canopy, thus more attention should be paid to the study of the attenuation of airflow in the outer layer of the canopy. The branches and leaves at a height of 0–0.3 m in the canopy can be pruned appropriately to increase the airflow velocity reaching the canopy and increase the disturbance to the inner canopy to improve the application effect, and to improve the light transmission and air permeability inside the canopy to some extent (Li et al., 2003; McDonald et al., 2013); (3) with an increase in the incoming flow velocity, the attenuation of airflow velocity inside the canopy is greater; therefore, the effect of enhancing the disturbance to the branches and leaves inside the canopy by increasing the incoming flow velocity will decrease with an increase in canopy depth; (4) there is a significant difference between the theoretical and experimental values of the airflow velocity of the outer canopy on the windward side when the flow velocity is larger. This is because the airflow affects the windward area of the outer canopy and subsequently reacts to the airflow, thereby forming an interactive coupling effect.

This study solves the issue of unclear airflow attenuation in the canopy, but certain limitations are still present. (1) only the pear canopy at the same growth period was selected as the research object to verify the accuracy of the model; the leaf area density canopy of the limit working conditions was not verified on other fruit trees. (2) because of the inconvenience in measuring the aerodynamic resistance parameters of the canopy, the canopy foliage was selected instead of the entire canopy. (3) although the influencing factors of the velocity decay factor k and the previously unknown state were sorted, resistance F is still an unassailable parameter, and the quantitative relationship between F and some easily measured parameters of the canopy needs to be further studied. (4) for different types of the canopy was not considered, the current study could not apply directly to commercial orchards based on different planting systems (open-vase, trellis, high-density, etc.).

Although the study has certain limitations, it theoretically reveals the attenuation law of the canopy airflow and its influencing factors. Future studies will track k-values at different growth stages of several representative pear trees; seek the relationship between k-values, canopy types, and growth periods; and establish a k-value database for pear trees. At the same time, different fruit tree varieties (such as apple trees, peach trees) and different planting systems (open-vase, trellis, high-density, etc.) will be selected to evaluate the applicability of the model. In addition, the optimal intra-canopy airflow velocity can be constructed according to the characteristics of the canopy structure, and the fan speed can be regulated in combination with the wind flow velocity attenuation model in the canopy.

## 5 Conclusions

This paper clarifies that the intra-canopy airflow velocity attenuation factor k in air-assisted spraying process for pear trees is related to the resistance F, the canopy leaf windward area s and the incoming flow velocity v_∗_, and constructs the model of airflow velocity attenuation. The model validation results reveal that the average accuracy of the model is around 80% under various working conditions.

As the incoming flow velocity changes the windward area of canopy leaves in the 0–0.3 m region, the greatest difference between the experimental and theoretical values of intra-canopy airflow occurs in this region. In this study, the 0.3 m-thick canopy was used as the research object to modify the model. For the different leaf area density canopy, the R^2^ of the theoretical airflow velocity and the actual airflow velocity is above 0.9.

The field experiment results show that the error between the theoretical airflow velocity and actual airflow velocity at any position in the canopy is within 0.7 m/s when the incoming velocity is 8m/s and 12m/s. This indicates that the airflow velocity attenuation model has good applicability in the orchards.

## Data availability statement

The original contributions presented in the study are included in the article/supplementary material. Further inquiries can be directed to the corresponding author.

## Author contributions

FZ and HS designed the study, performed the experiments. FZ and WQ wrote the manuscript and analyzed the data. XL and WQ suggested changes to the article. YC and GZ contributed to the concept of the study. All authors contributed to the article and approved the submitted version.

## Funding

This work was co-financed by the National Natural Science Foundation of China (Grant No. 32171905), the Fundamental Research Funds for the Central Universities (KYCYXT2022016), Jiangsu Agricultural Science and Technology Innovation Fund (CX203172), and the ‘Qinglan Project’ of Jiangsu Province (QLGC).

## Acknowledgments

Appreciations are given to the editor and reviewers of the Journal.

## Conflict of interest

The authors declare that the research was conducted in the absence of any commercial or financial relationships that could be construed as a potential conflict of interest.

## Publisher’s note

All claims expressed in this article are solely those of the authors and do not necessarily represent those of their affiliated organizations, or those of the publisher, the editors and the reviewers. Any product that may be evaluated in this article, or claim that may be made by its manufacturer, is not guaranteed or endorsed by the publisher.
